# Supplementary material for: Mammaglobin-A Expression Is Highly Specific for Tumors Derived from the Breast, the Female Genital Tract, and the Salivary Gland
Source: Diagnostics (Basel). 2023 Mar 22;13(6):1202. doi: 10.3390/diagnostics13061202 (PMC10047670; doi:10.3390/diagnostics13061202)
Supplement: Supplementary file 1 [file diagnostics-13-01202-s001.zip › Mammaglobin_suppl tab 1.pdf]

Reference

1 Kamanda S, Epstein JI, Osunkoya AO*, et al.* Poorly Differentiated Scrotal Carcinoma With Apocrine Immunophenotype. *Am J Dermatopathol*. 2022; **44**: 260-66.

2 Agaimy A, Ihrler S, Baneckova M*, et al.* HMGA2-WIF1 Rearrangements Characterize a Distinctive Subset of Salivary Pleomorphic Adenomas With Prominent Trabecular (Canalicular Adenoma-like) Morphology. *Am J Surg Pathol*. 2022; **46**: 190-99.

3 Maeshima Y, Osako T, Morizono H*, et al.* Metastatic ovarian cancer spreading into mammary ducts mimicking an in situ component of primary breast cancer: a case report. *J Med Case Rep*. 2021; **15**: 78.

4 Jackson J, Walker E, Bethune R, Bracey T, Mason C, Mandalia T. Extramammary Borderline Phyllodes Tumor Presenting as an Umbilical Mass. *Int J Surg Pathol*. 2021; **29**: 648-52.

5 Bishop JA, Nakaguro M, Whaley RD*, et al.* Oncocytic intraductal carcinoma of salivary glands: a distinct variant with TRIM33-RET fusions and BRAF V600E mutations. *Histopathology*. 2021; **79**: 338-46.

6 Palakkamanil MM, Mahmood MN, Chan A. Diagnostic and treatment challenges of a case of primary cutaneous signet-ring cell/histiocytoid carcinoma of the eyelid. *BMC Ophthalmol*. 2020; **20**: 410.

7 Kaleem A, Patel N, Alzahrani S, Hatoum H, Tursun R. Concurrent presence of secretory carcinoma and Warthin's tumor in ipsilateral parotid gland. *Oral Oncol*. 2020; **109**: 104691.

8 Soares CD, de Lima Morais TM, Carlos R*, et al.* Immunohistochemical expression of mammaglobin in salivary duct carcinomas de novo and salivary duct carcinoma ex pleomorphic adenoma. *Hum Pathol*. 2019; **92**: 59-66.

9 Nakaguro M, Urano M, Suzuki H*, et al.* Low-grade intraductal carcinoma of the salivary gland with prominent oncocytic change: a newly described variant. *Histopathology*. 2018; **73**: 314-20.

10 Woo HY, Choi EC, Yoon SO. Diagnostic Approaches for Salivary Gland Tumors with Secretory and Microcystic Features. *Head Neck Pathol*. 2018; **12**: 237-43.

11 Hu S, Hinson JL, Matnani R, Cibull ML, Karabakhtsian RG. Are the uterine serous carcinomas underdiagnosed? Histomorphologic and immunohistochemical correlates and clinical follow up in high-grade endometrial carcinomas initially diagnosed as high-grade endometrioid carcinoma. *Mod Pathol*. 2018; **31**: 358-64.

12 El Hag MI, Hag AM, Ha JP, Michael CW. Comparison of GATA-3, mammaglobin, GCDFP-15 expression in breast carcinoma in serous effusions: A cell-block micro-array study. *Pleura Peritoneum*. 2017; **2**: 143-48.

13 Baghai F, Yazdani F, Etebarian A, Garajei A, Skalova A. Clinicopathologic and molecular characterization of mammary analogue secretory carcinoma of salivary gland origin. *Pathol Res Pract*. 2017; **213**: 1112-18.

14 Alexiev BA, Jennings LJ, Samant S, Rao S. Oncocytic papillary cystadenoma with prominent mucinous differentiation of parotid gland: A case report. *Pathol Res Pract*. 2017; **213**: 1310-14.

15 Kusafuka K, Kawasaki T, Nakajima T, Sugino T. Carcinoma ex basal cell adenoma of the parotid gland: A report of an extremely rare case. *Pathol Int*. 2017; **67**: 355-60.

16 Shaoxian T, Baohua Y, Xiaoli X*, et al.* Characterisation of GATA3 expression in invasive breast cancer: differences in histological subtypes and immunohistochemically defined molecular subtypes. *J Clin Pathol*. 2017; **70**: 926-34.
17 Kandalaft PL, Simon RA, Isacson C, Gown AM. Comparative Sensitivities and Specificities of Antibodies to Breast Markers GCDFP-15, Mammaglobin A, and Different Clones of Antibodies to GATA-3: A Study of 338 Tumors Using Whole Sections. *Appl Immunohistochem Mol Morphol*. 2016; **24**: 609-14.
18 Said-Al-Naief N, Carlos R, Vance GH, Miller C, Edwards PC. Combined DOG1 and Mammaglobin Immunohistochemistry Is Comparable to ETV6-breakapart Analysis for Differentiating Between Papillary Cystic Variants of Acinic Cell Carcinoma and Mammary Analogue Secretory Carcinoma. *Int J Surg Pathol*. 2017; **25**: 127-40.
19 Hsieh MS, Lee YH, Chang YL. SOX10-positive salivary gland tumors: a growing list, including mammary analogue secretory carcinoma of the salivary gland, sialoblastoma, low-grade salivary duct carcinoma, basal cell adenoma/adenocarcinoma, and a subgroup of mucoepidermoid carcinoma. *Hum Pathol*. 2016; **56**: 134-42.
20 Hsieh MS, Jeng YM, Jhuang YL, Chou YH, Lin CY. Carbonic anhydrase VI: a novel marker for salivary serous acinar differentiation and its application to discriminate acinic cell carcinoma from mammary analogue secretory carcinoma of the salivary gland. *Histopathology*. 2016; **68**: 641-7.
21 Dyhdalo KS, Booth CN, Brainard JA*, et al.* Utility of GATA3, mammaglobin, GCDFP-15, and ER in the detection of intrathoracic metastatic breast carcinoma. *J Am Soc Cytopathol*. 2015; **4**: 218-24.
22 Wendroth SM, Mentrikoski MJ, Wick MR. GATA3 expression in morphologic subtypes of breast carcinoma: a comparison with gross cystic disease fluid protein 15 and mammaglobin. *Ann Diagn Pathol*. 2015; **19**: 6-9.
23 Serrano-Arevalo ML, Mosqueda-Taylor A, Dominguez-Malagon H, Michal M. Mammary analogue secretory carcinoma (MASC) of salivary gland in four Mexican patients. *Med Oral Patol Oral Cir Bucal*. 2015; **20**: e23-9.
24 Projetti F, Lacroix-Triki M, Serrano E*, et al.* A comparative immunohistochemistry study of diagnostic tools in salivary gland tumors: usefulness of mammaglobin, gross cystic disease fluid protein 15, and p63 cytoplasmic staining for the diagnosis of mammary analog secretory carcinoma? *J Oral Pathol Med*. 2015; **44**: 244-51.
25 Patel KR, Solomon IH, El-Mofty SK, Lewis JS, Jr., Chernock RD. Mammaglobin and S-100 immunoreactivity in salivary gland carcinomas other than mammary analogue secretory carcinoma. *Hum Pathol*. 2013; **44**: 2501-8.
