# Supplementary figures and images for: Mammaglobin-A Expression Is Highly Specific for Tumors Derived from the Breast, the Female Genital Tract, and the Salivary Gland
Source: Diagnostics (Basel). 2023 Mar 22;13(6):1202. doi: 10.3390/diagnostics13061202 (PMC10047670; doi:10.3390/diagnostics13061202)

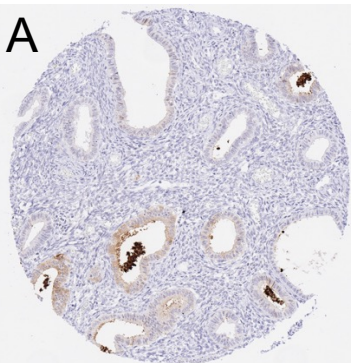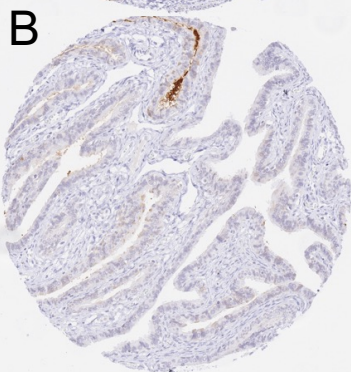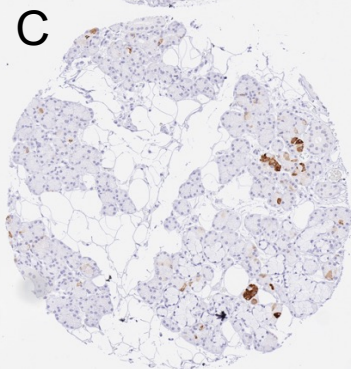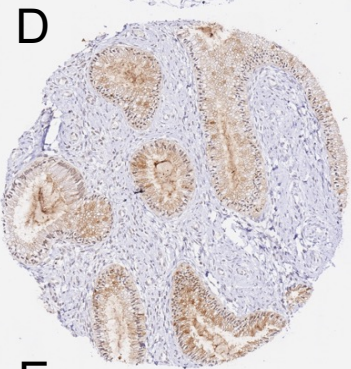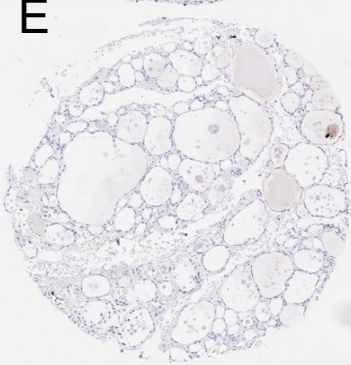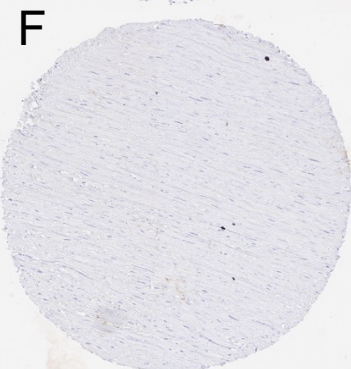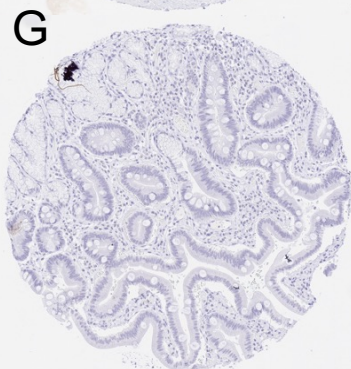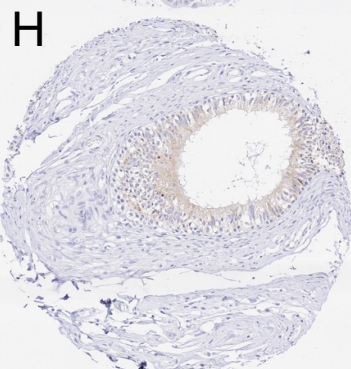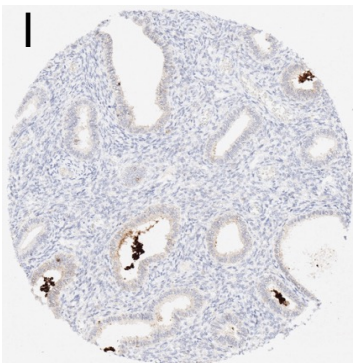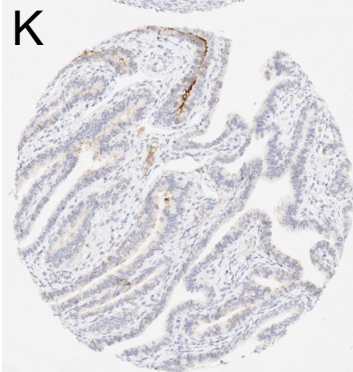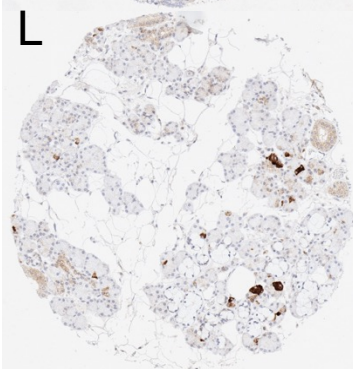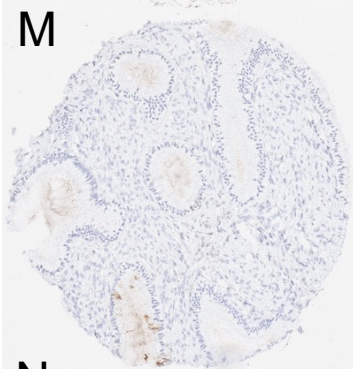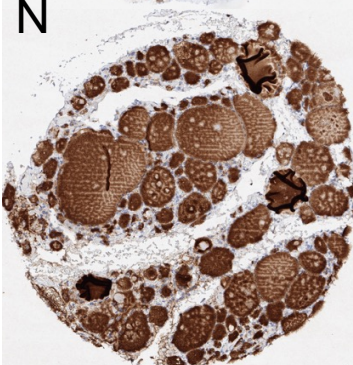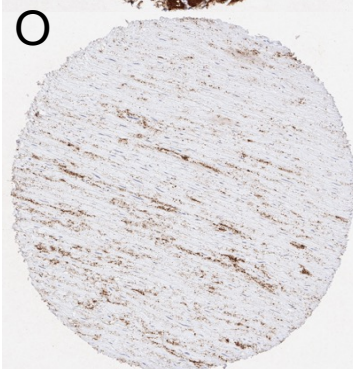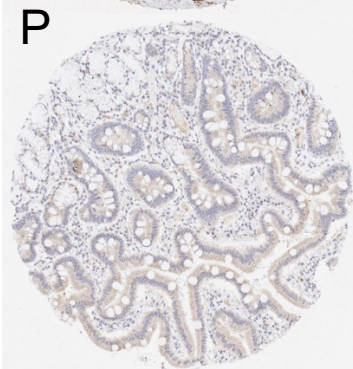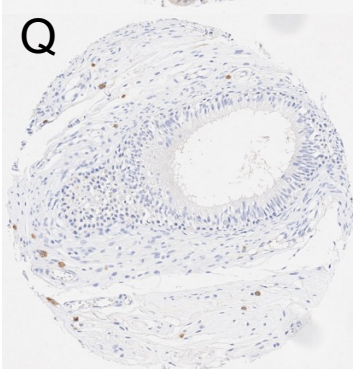

Supplement: Supplementary file 1 [file diagnostics-13-01202-s001.zip › Mammmaglobin A_suppl Figure 1.pdf]
